# Chronic Post-Concussion Neurocognitive Deficits. I. Relationship with White Matter Integrity

**DOI:** 10.3389/fnhum.2016.00035

**Published:** 2016-02-10

**Authors:** Jun Maruta, Eva M. Palacios, Robert D. Zimmerman, Jamshid Ghajar, Pratik Mukherjee

**Affiliations:** ^1^Brain Trauma FoundationNew York, NY USA; ^2^Department of Radiology and Biomedical Imaging, School of Medicine, University of California, San FranciscoSan Francisco, CA USA; ^3^Department of Radiology, Weill Cornell Medical CollegeNew York, NY USA; ^4^Department of Neurosurgery, Stanford UniversityStanford, CA USA

**Keywords:** eye movement, diffusion tensor imaging, mild traumatic brain injury, neuroimaging, neuropsychology, post-concussion syndrome

## Abstract

We previously identified visual tracking deficits and associated degradation of integrity in specific white matter tracts as characteristics of concussion. We re-explored these characteristics in adult patients with persistent post-concussive symptoms using independent new data acquired during 2009–2012. Thirty-two patients and 126 normal controls underwent cognitive assessments and MR-DTI. After data collection, a subset of control subjects was selected to be individually paired with patients based on gender and age. We identified patients’ cognitive deficits through pairwise comparisons between patients and matched control subjects. Within the remaining 94 normal subjects, we identified white matter tracts whose integrity correlated with metrics that indicated performance degradation in patients. We then tested for reduced integrity in these white matter tracts in patients relative to matched controls. Most patients showed no abnormality in MR images unlike the previous study. Patients’ visual tracking was generally normal. Patients’ response times in an attention task were slowed, but could not be explained as reduced integrity of white matter tracts relating to normal response timing. In the present patient cohort, we did not observe behavioral or anatomical deficits that we previously identified as characteristic of concussion. The recent cohort likely represented those with milder injury compared to the earlier cohort. The discrepancy may be explained by a change in the patient recruitment pool circa 2007 associated with an increase in public awareness of concussion.

## Introduction

The lack of a clear neurobiological explanation for cognitive deficits associated with late post-concussion symptoms is puzzling. On the one hand, it is understood that the chronic phase of traumatic brain injury represents axonal degeneration and neuronal cell death that may follow the acute injury. It is also understood that the initial susceptibility to injury due to a concussive impact is distributed unevenly within the brain (Ommaya and Gennarelli, 1974; Sabet et al., 2008). Accordingly, any tendency for damage in specific neural sites is expected to correspond to a trend in functional vulnerability, and thus quantification of neural damage at these specific sites may be used as a biomarker for neurocognitive dysfunction in concussion (Shenton et al., 2012; Eierud et al., 2014).

In our previous studies of patients with chronic post-concussion symptoms, we found associations between cognitive deficits, as identified through neurocognitive testing, and reduced integrity of white matter tracts underlying the prefrontal cortex, as identified by diffusion tensor imaging (DTI; Niogi et al., 2008; Maruta et al., 2010b). As a behavioral outcome of concussive brain injury, both chronically and acutely, we have characterized deficits in visual tracking of a predictably moving target (Maruta et al., 2010b, 2012). Moreover, in patients with chronic symptoms we have found these visual tracking deficits to be associated with reduced performance in other neurocognitive tests as well as with reduced white matter connectivity to the right prefrontal cortex (Maruta et al., 2010b). These findings supported the explanation for various symptoms associated with concussion as a result of predictive timing deficits caused by shearing injuries in the frontal white matter tracts (Ghajar and Ivry, 2008; Maruta et al., 2010a).

In contrast, while there may be a direct biological explanation for post-concussion symptoms for some patients, psychological processes may predominate in the development of post-concussion symptoms in other patients (King, 2003; Silver, 2014). Under these conditions, the initial biomechanical injury is regarded in relation to emerging symptoms as a precipitating event, rather than as an explanatory diagnosis. Ultimately the origin of cognitive or other problems and their perception must reside in some form within the brain, but to individually varying degrees, the link between the evolving problems and the initial biomechanical insult may become difficult to trace over time. This subject matter is considered in more depth in a companion paper (Maruta et al., 2016).

In this study, by combining anatomical and behavioral approaches as we have done previously (Maruta et al., 2010b), we examined independent new data acquired during the period of 2009–2012. We aimed to determine in this new cohort the extent to which performance differences between patients with persistent post-concussion symptoms and control subjects could be explained as reduced microstructural integrity of specific white matter tracts. As with the previous study, we focused on patients with persistent post-concussion symptoms since they had presumably developed stable neurological deficits.

## Materials and Methods

### Subjects

The sample consisted of 32 patients with a single isolated history of concussive injury to the head that occurred between 90 days and 5 years prior to testing (**Table 1**), and 126 control subjects without a history of brain injury. Recruitment for all subjects was limited to individuals between ages 18 and 55 and with at least 12 years of education. Patients must have had persistent symptoms believed to result from an isolated concussive injury at the time of recruitment, received documented medical attention at the time of injury, had posttraumatic amnesia at the time of injury, and had a loss of consciousness not exceeding 24 h in the period following the injury. In actuality, only three patients reported having had loss of consciousness longer than 1 h following the injury. The conditions for exclusion included a history of gross vision or hearing problems, a history of substance abuse, a history of neurological or psychiatric disorders, and current pregnancy. Subjects were recruited via flyers posted on college campuses and community centers in and around the New York City area, and newsletters of local brain injury-related organizations. Patients were also recruited via referrals from health professionals. Recruitment information for both groups was made available on the Brain Trauma Foundation website and in the newsletters of other local brain injury organizations. Potential subjects were screened before enrollment in the study following a protocol similar to the one used in the previous study (Maruta et al., 2010b), which included a modified Head Injury Symptoms Checklist (McLean et al., 1984). Although the patient recruitment effort in the previous study utilized referral from health professionals, in both studies the screening for initial inclusion was primarily based on subject self-report of diagnosis and symptoms since all subjects initiated contact to participate in the studies. How potential subjects had learned about the either study was not recorded.

**Table 1:** Patient demographics and summary of conventional clinical MRI findings.

Age (y)	Gender	Time since injury (mo)	Conventional MRI reading (Number of tracts with abnormally low FA)
19	Female	46.1	Normal (0)
19	Female	20.5	Focus of T2 hyperintensity in the left frontal white matter (0)
20	Female	19.2	Foci of T2 hyperintensity in the left subfrontal and parietal subcortical white matter (1)
22	Female	7.9	Foci of T2 hyperintensity in the left frontal subcortical white matter (0)
22	Female	8.7	Normal (0)
26	Female	26.5	Normal (0)
42	Female	12.8	Normal (0)
43	Female	21.8	Normal (0)
45	Female	51.8	Focal oval extra-axial T1 and T2 hyperintense structures adjacent to the left temporal lobe (0)
45	Female	13.4	Normal (0)
45	Female	15.1	Foci of T2 hyperintensity in predominantly in the right frontal subcortical white matter (1)
46	Female	20.1	Foci of T2 hyperintensity in the bilateral frontal lobes and external capsules (0)
49	Female	13.0	Normal (0)
49	Female	54.7	Normal (0)
51	Female	5.5	Multiple foci of white matter T2 hyperintensity consistent with microvascular ischemic change (0)
52	Female	24.8	Normal (0)
55	Female	34.0	Foci of T2 hyperintensity in the bilateral frontal and left parietal subcortical white matter (0)
18	Male	29.0	Normal (0)
19	Male	5.6	Normal (0)
20	Male	20.8	Normal (0)
20	Male	4.6	Normal (2)
20	Male	34.4	Focus of T2 hyperintensity in the right anterior subinsular white matter (0)
20	Male	10.2	Normal (0)
21	Male	12.8	Normal (0)
22	Male	9.2	Focus of T2 hyperintensity in the right frontal subcortical white matter (0)
24	Male	10.8	Normal (0)
28	Male	16.0	Foci of susceptibility hypointensity in the right frontal white matter (1)
37	Male	15.0	Normal (0)
47	Male	11.6	Normal (4)
51	Male	4.6	Focus of T2 hyperintensity in the left frontal subcortical white matter (2)
52	Male	26.9	Normal (0)
54	Male	33.3	Sulcal prominence. Foci of T2 hyperintensity in the right hemispheric supratentorial white matter with involvement of the body of the corpus callosum. Microbleeds in the right anterior temporal lobe and right side of the genu of the corpus callosum (0)

All in-person testing was conducted at the Citigroup Biomedical Imaging Center at Weill Cornell Medical College (WCMC) in New York, NY, USA between May 2009 and November 2012. The protocol was reviewed and approved by the WCMC Institutional Review Board. Written informed consent was obtained from all participants prior to data collection. Further clinical information was collected using the following measures: the Conners Adult ADHD Rating Scale – Self-Report: Short Version (CAARS-S:S) (Pearson, San Antonio, TX, USA); the PTSD Checklist – Civilian Version (National Center for PTSD, US Department of Veterans Affairs); the Center for Epidemiologic Studies Depression Scale (CES-D; Radloff, 1977); and the Brain Injury Screening Questionnaire (BISQ; Gordon et al., 2000). For control subjects, having a *T*-score of 75 or greater on the CAARS-S:S, having a score of 16 or greater on the CES-D, or not having a negative brain injury rating on the BISQ constituted additional criteria for exclusion from analysis. Of 140 control subjects tested eligibly, 135 completed MR scans, and finally the scan data from 126 subjects were deemed to be of an acceptable quality (see below). Of 33 patients tested eligibly, 32 completed MR scans, and their data were all deemed to be of an acceptable quality.

### Magnetic Resonance Imaging

Subjects underwent T1-, T2-, susceptibility-, and diffusion-weighted MR scans on a 3 Tesla GE Signa EXCITE scanner (GE Healthcare, Waukesha, WI, USA). High angular resolution data were acquired using 55 isotropically distributed diffusion-encoding directions at *b* = 1000 s/mm^2^ and one at b = 0 s/mm^2^, with 72 interleaved slices of 1.8 mm thickness, a 128 × 128 matrix that was zero-filled during reconstruction to 256 × 256, and a field of view (FOV) of 230 mm. The images had a resolution of 0.8 mm × 0.8 mm × 1.8 mm. The structural scan was an axial three-dimensional inversion-recovery fast spoiled gradient-recalled echo sequence (TE = 1.5 ms, TR = 6.3 ms, TI = 400 ms, flip angle = 15°) with a 256 × 256 matrix over a 230 mm FOV and 156 1.0-mm contiguous partitions.

All images were visually inspected for aberrant data. Data pre-processing and analysis were performed using the Diffusion Toolbox of the FMRIB Software Library (FSL 5.0) (Behrens et al., 2003; Smith et al., 2004). The diffusion images were first corrected for eddy current and motion artifacts and the registered images were skull-stripped using the Brain Extraction Tool (Smith, 2002). The global white matter integrity was assessed with fractional anisotropy (FA), mean diffusivity (MD), axial diffusivity (AD), and radial diffusivity (RD). After calculation of the FA map for each subject, we implemented a statistical analysis of the FA data using Tract-Based Spatial Statistics (Smith et al., 2006, 2007).

We focused on FA since we previously characterized reduced integrity of white matter tracts with this metric and associated it with behavioral deficits (Niogi et al., 2008; Maruta et al., 2010b). In an improvement to the previous manual region-of-interest approach, using FSL, data from all subjects were aligned to the standard space FMRIB58 adult FA template and averaged to create a study-specific FA template. The mean FA image was thinned to create a skeleton that represented the centers of all white matter tracts common to the present cohort of subjects. Segments of the FA skeleton were labeled according to the Johns Hopkins University ICBM-DTI-81 atlas (Mori et al., 2008). Forty-eight labels were created within FSL 5.0. Each subject’s aligned FA data were then projected onto this skeleton, and the averaged FA value within each labeled region of interest was used in further statistical analyses. Identified problems with ICBM-DTI-81 (Rohlfing, 2013) were addressed, in particular the left-right mirroring problem in the 2012 version, and the results were verified with known asymmetry in specific tracts of normal adults (Gong et al., 2005). Lastly, following our previous study (Maruta et al., 2010b), we identified white matter tracts in patients as damaged if the FA value associated with the tract label was less than the mean minus 2.5 times the standard deviation of the 126 control subjects.

### Behavioral Measures

Subjects underwent a neurocognitive testing battery that included a circular visual tracking test (Maruta et al., 2013), the Attention Network Test (ANT; Fan et al., 2002), the California Verbal Learning Test, 2nd edition (CVLT-II, Pearson), and the Wechsler Test of Adult Reading (WTAR, Pearson) among other tests. The WTAR was used to estimate the subjects’ (premorbid) full-scale IQ (Green et al., 2008). To provide a comparison to our previous study with an independent set of subjects (Maruta et al., 2010b), we focused on the data obtained from the visual tracking test, the ANT, and the CVLT-II.

#### Visual Tracking

The eye movement recording and analysis procedures are detailed in another publication (Maruta et al., 2013). Briefly, the visual tracking testing protocol was implemented on an integrated stimulus presentation-eye tracking apparatus (EyeLink CL, SR Research, Mississauga, ON, Canada) with which eye movements were recorded at 500 Hz while the subject’s head was stabilized. Prior to testing, it was verified that all subjects had normal or corrected-to-normal vision. The test stimulus was a target that moved clockwise in a circular trajectory of 10° radius at 0.4 Hz, which the subject was instructed to visually follow. The testing protocol including camera setup, calibration, practice and recorded runs, and associated instructions lasted approximately 5 min.

We utilized the continuous circular target motion because it is highly predictable. During visual tracking of a moving target, spatial, and temporal predictions are used to circumvent the neural delay required for the visuo-motor processing. In particular, the internally generated predictions must be synchronized to the external stimulus during continuous tracking. The stability of the gaze on the target was characterized by the variability of the instantaneous gaze positional error in the directions orthogonal and parallel to the target movement (SD of radial and tangential errors – SDRE, SDTE, respectively). As indicator of overall temporal accuracy, mean phase error was examined. We also characterized horizontal and vertical smooth pursuit velocity gain (H and V gains), the ratios between the smooth pursuit eye velocity and the target velocity in the horizontal and vertical direction, respectively.

#### Attention Control

The ANT is a computer-based test designed to delineate the efficiency of attention subcomponents by utilizing reaction time (RT) measures and various cue and target stimulus combinations (Fan et al., 2002). The alerting, orienting, and executive effects were measured as the median RT of no-cue trials minus the median RT of double cue trials, the median RT of central cue trials minus the median RT of spatial cue trials, and the median RT of incongruent flanker trials minus the median RT of congruent flanker trials, respectively. The overall accuracy across cue conditions and the overall processing efficiency, measured as the median of raw RTs for accurate responses across cue conditions, were recorded.

#### Verbal Working Memory

The CVLT-II is a verbal learning and memory test, in which the examinee is read a list of words and asked to recall them across a series of trials. The total recall discriminability and recognition discriminability indices of the CVLT-II are reported to vary meaningfully with the degree of severity of TBI (Jacobs and Donders, 2007). Thus, we used these indices for the comparisons between patients and control subjects. In addition, we used the raw counts of the long delay free recall trial since this index was previously shown to correlate with the integrity of the uncinate fasciculus in patients with persistent symptoms after concussion (Niogi et al., 2008).

### Statistical Analysis

A normal control subject was matched to each of the 32 patients by gender and age criteria. We matched subjects based on gender and age because regional FA values are influenced by these factors (Hsu et al., 2008; Inano et al., 2011). Pairwise comparisons of individual characteristics of patients and matched controls were made using paired *t*-tests (Niven et al., 2012). For the comparisons of demographics, performance, and global DTI metrics, the null hypothesis was that the scores for patients and controls were no different. The null hypothesis for the regional FA comparisons was that the scores for patients were lower than controls as indicative of reduced white matter integrity. A two-sample *t*-test was used to test for a difference in demographic variables between the normal subjects who were matched to patients and those remaining, who were not matched to patients. Using the latter pool of 94 normal subjects, we explored relationships between integrity of white matter tracts and our specific neurocognitive metrics. Correlations between regional FA values and cognitive test performance in unmatched normal subjects were tested using Pearson’s approach. The alpha level was set at 0.05. Correlations in the unmatched normal subjects were examined to select brain regions to form a basis for hypothesis-driven comparisons between patients and matched controls, and thus were tested without adjustments for multiple comparisons. We used a chi-squared test to compare the frequencies of normal and abnormal findings from conventional MRI against those from our previous study (Maruta et al., 2010b).

One patient did not undergo the CVLT-II or the WTAR because these tests were used as part of the evaluation at the referring medical facility. One control subject who was matched to a patient failed to make a sufficient number of correct responses on the ANT, which resulted in incomplete scoring of the test for this subject. Technical errors accounted for missing test scores for one unmatched normal subject on the visual tracking test. We report the results of analyses based on all observed data without regard for the missing data, i.e., without case deletion or replacing missing data with substituted values.

## Results

### Study Population

Within the 32 case-control pairs, the genders of the patients and controls were completely matched by design (17 female pairs, **Table 2**). The patients were on average just 0.3 years younger than the controls, and this difference was not statistically significant (|*t*(31)| = 1.77, *p* = 0.086). We also compared the years of education completed by the subjects and WTAR-estimated IQ to ensure that the matching procedure did not create a systematic bias in other background measures. We found no statistical difference in the years of education (|*t*(31)| = 1.31), *p* = 0.20) or estimated IQ scores (|*t*(30)| = 0.31, *p* = 0.76).

**Table 2:** Case-control matching.

	Patient	Control	Pairwise difference (Patient – Control)
Gender	47% male	47% male	–
Age (yr)	34.5 (13.8)	34.8 (13.5)	– 0.3 (1)
Time since injury (mo)	20.0 (13.1)	–	–
Education (yr)	15.2 (2.5)	15.8 (2.1)	– 0.6 (2.7)
Estimated IQ	109.2 (7.9)^∗^	109.6 (8.8)	– 0.7 (11.8)^∗^

*Entry values indicate mean (SD). ^∗^N = 31 instead of 32.*

Within the remaining 94 normal subjects (50 females) who were not matched to a patient, the mean (SD) of age was 36.5 (9.6), years of education was 17.4 (2.4), and WTAR-estimated IQ was 110.3 (7.5). This subgroup of subjects on average had more years of education than the patient-matched normal subgroup [*t*(124) = 3.49, *p* < 0.001], but the educational attainments characterizing the matched pairs were represented by both subgroups. Thus, the matched controls were not comprised of exceptions within the parent set, and inferences made from the unmatched normal subjects were deemed applicable to comparisons between matched pairs.

### Case-Control Performance Differences

**FIGURE 1:**
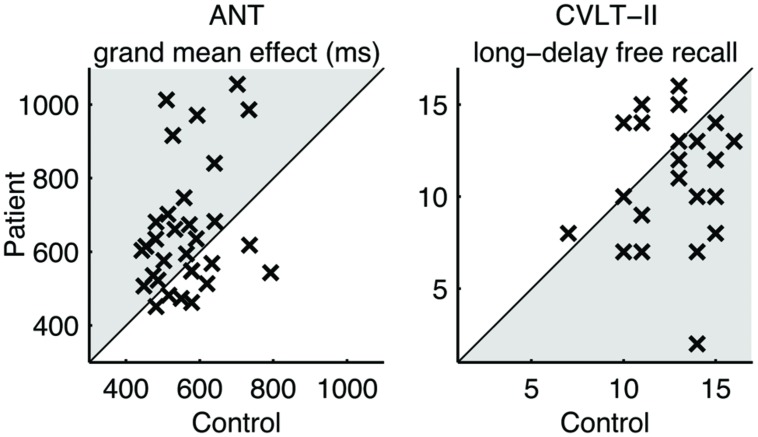
**Pairwise comparison of performance.** Each cross (×) represents a case-control pair. Crosses in the shaded areas indicate the poorer performance by patients.

**Table 3** summarizes the performance characteristics of patients and matched controls. Notably, the patients performed no worse than controls in the visual tracking task unlike the finding in our previous study (Maruta et al., 2010b). On the ANT, the patients indicated a general slowing of response times (**Figure 1**) but no deficit in any of the specific attention subcomponents. On the CVLT-II, the patients recalled fewer words on the long-delay free recall task, but indicated no differences in the standardized discriminability scores.

**Table 3:** Performance characteristics of patients and matched controls.

Performance metrics	Patient Mean (*SD*)	Control Mean (*SD*)	Paired *t*-test *p*-value
Visual tracking (32 pairs)			
SDRE (°va)	0.76 (0.30)	0.66 (0.23)	0.18
SDTE (°va)	1.01 (0.59)	0.95 (0.67)	0.73
Mean phase (°p)	– 0.32 (3.16)	– 0.30 (3.02)	0.98
H gain	0.90 (0.06)	0.92 (0.06)	0.13
V gain	0.79 (0.10)	0.81 (0.11)	0.45
ANT (31 pairs)			
Alerting effect (ms)	38 (35)	42 (25)	0.58
Orienting effect (ms)	51 (29)	40 (27)	0.17
Conflict effect (ms)	157 (78)	131 (53)	0.11
Grand mean effect (ms)	657 (169)	565 (88)	**0.005**
Accuracy (%)	96 (4)	97 (3)	0.54
CVLT-II (31 pairs)			
Tot. recall discriminability	0.10 (1.08)	0.45 (0.78)	0.13
Tot. recognition discriminability	– 0.02 (0.83)	0.31 (0.74)	0.098
Long-delay free recall	11.1 (3.0)	12.7 (2.1)	**0.019**

*Group means with SD and the results of paired t-tests are shown. Bold typeface indicates p < 0.05.*

### Identification of White Matter Regions of Interest

There was no statistical difference in the four global white matter DTI metrics in paired comparisons between patients and matched controls (|*t*(31)| < 1.54, *p* > 0.13). On average, the patients had FA, MD, AD, and RD values of 0.50, 0.72 × 10^–3^, 1.16 × 10^–3^, and 0.51 × 10^–3^, respectively, while the control subjects had 0.50, 0.72 × 10^–3^, 1.16 × 10^–3^, and 0.50 × 10^–3^, respectively.

Although the metrics of global white matter characteristics did not provide an explanation for the above-found reduced performance in patients, such an explanation may be found in regional white matter characteristics. To form a basis for hypothesis-driven comparisons, we first identified white matter tracts that influence neurocognitive performance in such a way that their reduced integrity may explain the observed performance differences. So as to identify such white matter tracts, regional FA values were correlated with pertinent performance metrics in an independent sample of normal subjects.

**FIGURE 2:**
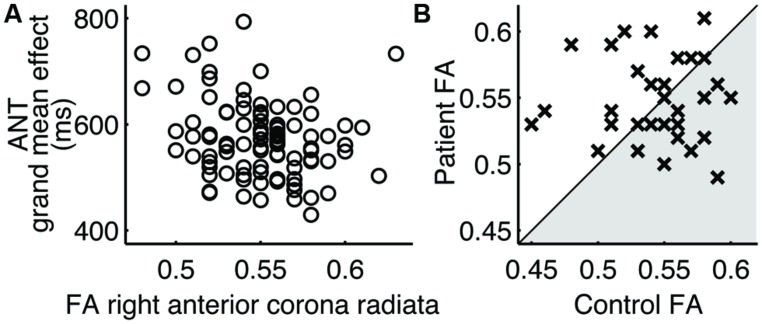
**Right anterior corona radiata. (**A**) Correlation to the ANT grand mean effect in 94 normal subjects. (B) Pairwise comparison of FA values.** Each cross (×) represents a case-control pair. Crosses in the shaded area indicate the lower FA value for patients.

Of the two performance metrics that indicated differences between patients and controls, the grand mean effect on the ANT, but not the long-delay free recall score on CVLT-II, was found to correlate with the FA values of white matter tracts (**Table 4**). The identified white matter tracts included the right anterior corona radiata (**Figure 2A**), for which we previously found low FA values commonly associated with patients in another subject cohort (Maruta et al., 2010b).

**Table 4:** Identified white matter regions and pairwise comparison of FA.

	Correlation (94 normal subjects)		Comparison of FA values (31 matched pairs)
Identified regions	*r*-value	*p*-value	Paired *t*-test *p*-value
Anterior corona radiata – right	– 0.24	**0.021**	0.80
Cerebral peduncle – right	– 0.25	**0.017**	0.92
Fornix	– 0.31	**0.0025**	0.83
Posterior thalamic radiation – left	– 0.27	**0.0075**	0.96
Retrolenticular internal capsule – left	– 0.23	**0.028**	0.99
Sagittal stratum – left	– 0.25	**0.014**	0.99
Sagittal stratum – right	– 0.26	**0.013**	≅1.0
Superior longitudinal fasciculus – left	– 0.21	**0.039**	0.95

*The correlations for the listed white matter regions are to the ANT grand mean effect. Bold typeface indicates p < 0.05.*

### Case-Control Regional FA Differences

The regional FA values in patients were no smaller than those in controls in paired comparisons (**Table 4**; **Figure 2B**). In fact, the mean FA values in these regions were somewhat larger in patients, contrary to the hypothesis. The mean FA values for patients and controls, respectively, in the right anterior corona radiata were 0.55 and 0.54 (|*t*(31)| = 0.84), the right cerebral peduncle were both 0.77 (|*t*(31)| = 1.43), the fornix were 0.65 and 0.62 (|*t*(31)| = 0.99), the left posterior thalamic radiation were 0.71 and 0.69 (|*t*(31)| = 1.76), the left retrolenticular internal capsule were 0.69 and 0.67 [(*t*(31) = 2.52], the left sagittal stratum were 0.64 and 0.62 (|*t*(31)| = 2.36), the right sagittal stratum were 0.64 and 0.62 (|*t*(31)| = 2.81), and the left superior longitudinal fasciculus were 0.59 and 0.57 (|*t*(31)| = 1.68).

### Comparison to Previous Patient Cohort

In the present study, 19 of the 32 patients (59.4%) had normal conventional MRI readings and none (0%) showed a contusion (**Table 1**). In contrast, in the previous study (Maruta et al., 2010b), 8 of the 17 patients (47.1%) had normal readings and 3 (17.6%) showed a contusion, both numbers indicating that the previous cohort included more severely injured patients. A chi-squared test showed that the relative frequencies of normal findings, abnormal findings without a contusion, and abnormal findings with a contusion in the present patient cohort were significantly different from those in the previous one [χ_(2)_^2^ = 6.94, *p* = 0.031].

In 26 of the 32 patients, no damage was identified in any of the 48 white matter tracts using DTI-FA measures (**Table 1**, also see Supplementary Table S1). Of the six patients who were found to have damage in the white matter, only three had damage in multiple tracts. In the previous study (Maruta et al., 2010b), even though only 11 white matter tracts were chosen for analysis, 13 of 17 patients were found to have damage in at least one tract, and seven of them had damage in multiple tracts. The contrast again indicated that the previous cohort included more severely injured patients.

## Discussion

In the present cohort of patients with persistent post-concussion symptoms, except for some non-specific cognitive deficits, we did not observe behavioral or anatomical deficits that we previously identified as characteristic of concussion. Specifically, these characteristics were deficits in predictive timing as demonstrated through quantification of predictive visual tracking performance (Maruta et al., 2010b, 2012, 2014) and reduced regional FA in DTI that correlated with specific cognitive deficits (Niogi et al., 2008; Maruta et al., 2010b). We consider our previous studies to be well-founded since large anticipatory saccades, a hallmark of impaired predictive timing, were the most prominent feature of visual tracking deficits in two independent patient cohorts representing acute and chronic stages of concussive injury (Maruta et al., 2010b, 2012, 2014). The structure-function link that we identified in the previous patient cohorts (Niogi et al., 2008; Maruta et al., 2010b) is also consistent with a frontal vulnerability to concussion (Eierud et al., 2014). Thus, the general lack of these characteristics in the present patient cohort needs explanation.

In one particular previous study (Maruta et al., 2010b), we utilized similar enrollment criteria, standardized tests, and analytical approaches to those in the present study. Both the present and the previous studies share a common limitation of being a cross-sectional study of a patient population with a great heterogeneity in terms of etiology and severity of the original trauma and patterns of symptom development. The behavior and DTI results for the controls were comparable between the two studies. Yet, only in the previous study did we find a clear difference between patients and controls, which is not explicable by the sample size difference of the studies. Although comparisons of concussion-related studies can be confounded by methodological inconsistencies (Eierud et al., 2014; Kristman et al., 2014), the constants between our two studies may render the comparison more amenable to interpretation. One key difference between the two studies is the time period during which they were conducted. The testing for the present study took place between 2009 and 2012, while the testing for the previous study took place between 2005 and 2006.

The difference in the time period of the two studies is critical because there was a dramatic increase in the rate of *reported* incidents of concussion around the time when recruitment for the older study was concluded (Lincoln et al., 2011; Cameron et al., 2012; Rosenthal et al., 2014; Marin et al., 2014). This increase occurred concurrently to enactment of new state laws in the U.S. and recommendations by various organizations, as well as increased media coverage on concussion (CDC, 2013; Guskiewicz et al., 2004; Ellenbogen et al., 2010); therefore, the apparent improvement in the rate of concussion detection is likely related to increased awareness of concussion (Lincoln et al., 2011; Marin et al., 2014; Rosenthal et al., 2014). Improved identification of concussion may also have resulted in an increase in subsequent pursuit of treatment for symptoms presumed to be associated with concussion even though these symptoms are not recognized to be unique to concussion (Iverson and Lange, 2003; Hoge et al., 2008; Ettenhofer and Barry, 2012). While these changes reflect a positive development in terms of public health, a fundamental change may have also occurred in the patient population at large, resulting in an expansion of the recruitment pool for patient-based studies to include the milder end of the spectrum of concussion or those who experienced a head impact without a brain injury. The possibility that the current patient cohort represented those with mild concussion is supported by the differences in MRI findings from our two studies. However, this explanation remains a speculation because the comparison of our two studies only provides an anecdotal record rather than a proof.

The present study lends support to the notion that, in many patients suffering from persistent symptoms long after a concussion, the original injury may contribute to the symptoms only indirectly (King, 2003; Silver, 2014). The nature of this contribution is not clear. To be cautious, however, while DTI may be currently the best *in vivo* method to detect structural abnormalities in human brains (Shenton et al., 2012), newer more sensitive diffusion MRI technologies with improved image acquisition and tissue modeling may find more subtle white matter pathology where DTI fails (Setsompop et al., 2012; Zhang et al., 2012). It is nevertheless unlikely that all symptoms and functional deficits can be characterized with structural attributes measurable at the voxel level; thus, a lack of imaging finding may not be automatically equated with the absence of injury.

It is likewise unclear whether the absence of visual tracking predictive timing deficits that we previously observed in patients with both acute and chronic stages of concussive injury (Maruta et al., 2010b, 2012, 2014) represents recovery or compensation in the present patient cohort. In a recent study, however, an independent group of patients with persistent post-concussion symptoms similarly had normal visual tracking performance but had abnormal blood oxygen level-dependent activity in functional MRI while performing the task (Astafiev et al., 2015), suggesting a possible functional compensation for altered neural circuits. Still, there may be a limit in the effectiveness of such functional compensation, and latent deficits may be revealed under increased physiological or cognitive stress (Temme et al., 2013; Maruta et al., 2016).

In summary, in the present cohort of patients from 2009 to 2012 with persistent post-concussion symptoms, we did not observe deficits in predictive timing or any other specific attention subcomponent. Nor were we able to explain the otherwise observed performance differences between patients and control subjects as reduced integrity in specific white matter tracts. Our present finding highlights the need to elucidate the nature of the original injury’s contribution to the symptoms in patients suffering from persistent symptoms long after even a mild concussion.

## Author Contributions

JM, JG, and PM designed experiments and oversaw data collection and analysis. EP, RZ, and PM conducted neuroradiological analyses. JM conducted statistical analyses. All authors contributed to the interpretation of data and to drafting and revising the work.

## Conflict of Interest Statement

JG is director of Sync-Think, Inc. and holds U.S. patent 7,384,399. JM holds stock option in Sync-Think. The authors declare that the research was conducted in the absence of any other commercial or financial relationships that could be construed as a potential conflict of interest.
