# Supplementary material for: Chronic Post-Concussion Neurocognitive Deficits. I. Relationship with White Matter Integrity
Source: Front Hum Neurosci. 2016 Feb 10;10:35. doi: 10.3389/fnhum.2016.00035 (PMC4748060; doi:10.3389/fnhum.2016.00035)
Supplement: Supplementary file 1 [file Table_S1.PDF]

Supplementary Material:

Chronic post-concussion neurocognitive deficits. I. Relationship with white matter integrity

Jun Maruta*, Eva M. Palacios, Robert D. Zimmerman, Jamshid Ghajar, Pratik Mukherjee*

**Correspondence:*
Jun Maruta
jmaruta@braintrauma.org
Pratik Mukherjee
pratik@radiology.ucsf.edu

Supplementary Table S1 | FA values of 48 regions of interest in patients.

Age	Gender	Time since injury (mo)	ACR-L	ACR-R	ALIC-L	ALIC-R	BCC	CGC-L	CGC-R	CGH-L	CGH-R	CP-L	CP-R	CST-L	CST-R	EC-L	EC-R	FX	FXST-L	FXST-R	GCC	ICP-L	ICP-R	MCP	ML-L	ML-R	PCR-L	PCR-R	PCT	PLIC-L	PLIC-R	PTR-L	PTR-R	RLIC-L	RLIC-R	SCC	SCP-L	SCP-R	SCR-L	SCR-R	SFO-L	SFO-R	SLF-L	SLF-R	SS-L	SS-R	TAP-L	TAP-R	UNC-L	UNC-R	Abnormally low FA count
19	F	46.1	0.50	0.51	0.66	0.64	0.73	0.66	0.67	0.66	0.74	0.77	0.76	0.67	0.67	0.54	0.52	0.51	0.63	0.60	0.75	0.61	0.64	0.65	0.73	0.71	0.60	0.54	0.63	0.73	0.69	0.69	0.69	0.69	0.65	0.86	0.74	0.71	0.55	0.52	0.58	0.55	0.60	0.56	0.69	0.62	0.65	0.66	0.51	0.61	0
19	F	20.5	0.58	0.56	0.70	0.67	0.76	0.71	0.69	0.69	0.68	0.81	0.79	0.70	0.69	0.60	0.57	0.63	0.68	0.67	0.76	0.62	0.63	0.65	0.69	0.68	0.58	0.53	0.64	0.76	0.73	0.72	0.73	0.69	0.68	0.86	0.74	0.77	0.58	0.53	0.61	0.57	0.59	0.57	0.66	0.64	0.55	0.65	0.57	0.63	0
20	F	19.2	0.56	0.49	0.67	0.64	0.75	0.69	0.65	0.65	0.61	0.79	0.79	0.67	0.69	0.51	0.51	0.67	0.65	0.61	0.75	0.59	0.60	0.61	0.65	0.69	0.58	0.53	0.54	0.72	0.72	0.69	0.68	0.70	0.64	0.88	0.76	0.76	0.51	0.49	0.60	0.53	0.54	0.53	0.59	0.63	0.69	0.68	**0.44**	0.52	1
22	F	7.9	0.60	0.59	0.69	0.67	0.76	0.74	0.71	0.70	0.72	0.78	0.79	0.64	0.66	0.60	0.58	0.70	0.67	0.68	0.77	0.64	0.63	0.64	0.66	0.68	0.55	0.55	0.58	0.74	0.70	0.71	0.71	0.68	0.68	0.86	0.75	0.74	0.57	0.54	0.66	0.61	0.60	0.58	0.66	0.65	0.69	0.64	0.54	0.64	0
22	F	8.7	0.57	0.53	0.67	0.68	0.78	0.70	0.65	0.70	0.67	0.80	0.81	0.70	0.66	0.58	0.55	0.75	0.73	0.69	0.79	0.67	0.67	0.64	0.66	0.66	0.57	0.52	0.61	0.77	0.73	0.74	0.73	0.70	0.67	0.85	0.71	0.71	0.57	0.52	0.64	0.63	0.60	0.58	0.69	0.68	0.78	0.65	0.64	0.60	0
26	F	26.5	0.57	0.53	0.67	0.67	0.74	0.70	0.63	0.74	0.66	0.81	0.80	0.69	0.70	0.54	0.51	0.61	0.64	0.62	0.78	0.65	0.65	0.63	0.66	0.66	0.56	0.51	0.61	0.77	0.74	0.71	0.69	0.67	0.67	0.85	0.73	0.69	0.56	0.52	0.54	0.59	0.58	0.52	0.63	0.63	0.70	0.65	0.55	0.56	0
42	F	12.8	0.60	0.60	0.67	0.67	0.75	0.67	0.65	0.71	0.69	0.79	0.78	0.70	0.69	0.53	0.55	0.76	0.69	0.70	0.81	0.66	0.63	0.62	0.66	0.64	0.59	0.54	0.57	0.74	0.71	0.69	0.69	0.68	0.63	0.84	0.71	0.69	0.55	0.50	0.60	0.61	0.60	0.58	0.63	0.64	0.56	0.63	0.56	0.59	0
43	F	21.8	0.53	0.51	0.65	0.64	0.73	0.66	0.63	0.62	0.60	0.76	0.76	0.65	0.66	0.53	0.51	0.67	0.66	0.61	0.76	0.63	0.62	0.63	0.68	0.64	0.53	0.54	0.60	0.72	0.68	0.69	0.67	0.63	0.63	0.85	0.68	0.70	0.53	0.49	0.54	0.54	0.56	0.55	0.63	0.63	0.54	0.69	0.53	0.50	0
45	F	51.8	0.56	0.51	0.66	0.63	0.75	0.73	0.70	0.66	0.64	0.76	0.76	0.66	0.64	0.57	0.55	0.51	0.71	0.61	0.76	0.62	0.63	0.65	0.65	0.65	0.62	0.55	0.54	0.82	0.74	0.74	0.73	0.77	0.70	0.88	0.67	0.67	0.55	0.52	0.61	0.64	0.61	0.60	0.64	0.65	0.69	0.73	0.57	0.58	0
45	F	13.4	0.57	0.54	0.68	0.67	0.75	0.72	0.64	0.66	0.68	0.77	0.76	0.67	0.64	0.54	0.52	0.70	0.65	0.66	0.76	0.66	0.65	0.64	0.69	0.64	0.59	0.55	0.57	0.74	0.74	0.72	0.71	0.68	0.67	0.89	0.75	0.72	0.55	0.52	0.58	0.58	0.59	0.55	0.62	0.62	0.72	0.64	0.50	0.62	0
45	F	15.1	0.54	0.54	0.63	0.64	0.73	0.65	0.63	0.68	0.77	0.77	0.78	0.62	0.60	**0.49**	0.52	0.72	0.69	0.71	0.75	0.65	0.64	0.64	0.66	0.63	0.53	0.52	0.56	0.72	0.72	0.66	0.70	0.67	0.66	0.85	0.72	0.72	0.53	0.52	0.58	0.60	0.54	0.56	0.64	0.66	0.68	0.63	0.55	0.51	1
46	F	20.1	0.59	0.60	0.67	0.65	0.76	0.68	0.66	0.60	0.65	0.78	0.75	0.64	0.63	0.56	0.55	0.73	0.64	0.65	0.80	0.64	0.63	0.64	0.66	0.64	0.56	0.55	0.54	0.71	0.68	0.73	0.74	0.66	0.70	0.87	0.72	0.70	0.54	0.51	0.61	0.56	0.60	0.59	0.65	0.70	0.62	0.67	0.52	0.58	0
49	F	13.0	0.55	0.57	0.68	0.66	0.76	0.73	0.68	0.68	0.67	0.78	0.78	0.66	0.66	0.55	0.55	0.71	0.71	0.69	0.79	0.65	0.64	0.63	0.68	0.69	0.53	0.55	0.57	0.73	0.70	0.73	0.71	0.68	0.67	0.85	0.74	0.74	0.55	0.53	0.61	0.60	0.59	0.57	0.67	0.67	0.68	0.70	0.57	0.55	0
49	F	54.7	0.54	0.53	0.66	0.67	0.76	0.73	0.67	0.67	0.72	0.80	0.78	0.69	0.67	0.54	0.52	0.64	0.68	0.64	0.78	0.65	0.66	0.63	0.68	0.67	0.57	0.56	0.60	0.72	0.71	0.71	0.70	0.66	0.63	0.87	0.76	0.74	0.55	0.52	0.67	0.60	0.62	0.59	0.59	0.60	0.61	0.72	0.57	0.66	0
51	F	5.5	0.56	0.54	0.64	0.66	0.72	0.66	0.63	0.68	0.72	0.77	0.75	0.67	0.67	0.54	0.52	0.67	0.68	0.66	0.74	0.65	0.64	0.64	0.68	0.65	0.55	0.53	0.58	0.73	0.70	0.64	0.64	0.67	0.64	0.85	0.75	0.76	0.53	0.50	0.58	0.57	0.58	0.56	0.64	0.64	0.71	0.66	0.49	0.61	0
52	F	24.8	0.59	0.58	0.68	0.65	0.76	0.73	0.67	0.66	0.66	0.80	0.79	0.63	0.63	0.56	0.54	0.74	0.70	0.67	0.80	0.70	0.65	0.61	0.67	0.66	0.53	0.56	0.53	0.72	0.71	0.73	0.72	0.68	0.67	0.87	0.72	0.69	0.55	0.51	0.61	0.62	0.56	0.59	0.63	0.65	0.68	0.71	0.57	0.57	0
55	F	34.0	0.62	0.59	0.68	0.66	0.77	0.70	0.64	0.72	0.75	0.83	0.82	0.74	0.69	0.57	0.54	0.73	0.72	0.65	0.78	0.65	0.69	0.69	0.74	0.71	0.57	0.55	0.61	0.79	0.76	0.74	0.75	0.77	0.72	0.89	0.77	0.73	0.58	0.54	0.63	0.61	0.63	0.61	0.71	0.67	0.69	0.69	0.59	0.69	0
18	M	29.0	0.56	0.58	0.70	0.67	0.78	0.72	0.68	0.77	0.71	0.78	0.77	0.66	0.72	0.54	0.54	0.45	0.62	0.63	0.74	0.59	0.61	0.64	0.67	0.66	0.60	0.57	0.59	0.73	0.70	0.67	0.67	0.71	0.66	0.88	0.81	0.74	0.63	0.53	0.71	0.59	0.61	0.56	0.63	0.61	0.79	0.68	0.47	0.56	0
19	M	5.6	0.61	0.58	0.69	0.69	0.76	0.76	0.72	0.74	0.73	0.81	0.79	0.73	0.68	0.60	0.60	0.63	0.70	0.66	0.82	0.70	0.71	0.65	0.68	0.68	0.60	0.59	0.61	0.77	0.75	0.74	0.72	0.74	0.73	0.87	0.72	0.72	0.59	0.55	0.55	0.59	0.64	0.61	0.71	0.68	0.82	0.73	0.52	0.61	0
20	M	20.8	0.56	0.56	0.67	0.62	0.77	0.72	0.66	0.70	0.69	0.76	0.77	0.68	0.64	0.55	0.53	0.69	0.65	0.67	0.76	0.66	0.65	0.64	0.70	0.68	0.54	0.54	0.61	0.74	0.72	0.73	0.73	0.69	0.68	0.88	0.73	0.73	0.54	0.52	0.56	0.55	0.58	0.57	0.66	0.63	0.60	0.68	0.50	0.58	0
20	M	4.6	0.51	0.50	0.65	0.63	0.71	0.68	0.63	0.60	**0.59**	0.74	0.76	0.61	0.59	0.51	0.49	0.54	0.61	0.58	0.73	0.64	0.63	0.61	0.67	0.65	0.51	0.52	0.57	0.71	0.68	0.69	0.68	0.68	0.65	0.85	0.72	0.69	0.51	0.49	0.55	0.59	0.54	0.55	0.63	0.62	0.52	0.59	0.48	**0.48**	2
20	M	34.4	0.60	0.55	0.66	0.65	0.77	0.70	0.66	0.63	0.66	0.81	0.81	0.69	0.68	0.58	0.55	0.56	0.62	0.61	0.79	0.62	0.62	0.64	0.71	0.72	0.60	0.60	0.57	0.78	0.75	0.76	0.73	0.70	0.69	0.91	0.74	0.71	0.62	0.54	0.64	0.64	0.60	0.58	0.65	0.67	0.58	0.67	0.62	0.66	0
20	M	10.2	0.55	0.56	0.63	0.62	0.74	0.72	0.69	0.64	0.63	0.76	0.77	0.65	0.61	0.54	0.55	0.71	0.62	0.64	0.79	0.66	0.62	0.60	0.68	0.66	0.57	0.54	0.55	0.70	0.68	0.70	0.68	0.67	0.62	0.84	0.70	0.70	0.54	0.48	0.55	0.58	0.58	0.56	0.64	0.63	0.58	0.65	0.55	0.62	0
21	M	12.8	0.62	0.61	0.70	0.68	0.76	0.72	0.69	0.72	0.70	0.80	0.80	0.65	0.63	0.56	0.55	0.70	0.66	0.61	0.82	0.68	0.64	0.63	0.70	0.67	0.58	0.55	0.59	0.77	0.75	0.74	0.71	0.73	0.68	0.86	0.71	0.72	0.58	0.54	0.63	0.63	0.62	0.60	0.68	0.66	0.78	0.70	0.56	0.61	0
22	M	9.2	0.55	0.53	0.65	0.63	0.72	0.71	0.66	0.66	0.65	0.77	0.75	0.61	0.61	0.55	0.53	0.64	0.64	0.62	0.75	0.63	0.63	0.62	0.61	0.61	0.57	0.55	0.53	0.73	0.71	0.68	0.69	0.75	0.69	0.86	0.69	0.73	0.55	0.50	0.56	0.52	0.59	0.56	0.61	0.62	0.75	0.73	0.50	0.52	0
24	M	10.8	0.57	0.53	0.65	0.63	0.71	0.69	0.63	0.72	0.68	0.75	0.74	0.62	0.63	0.55	0.54	0.64	0.64	0.62	0.75	0.67	0.69	0.63	0.64	0.63	0.54	0.52	0.58	0.71	0.70	0.71	0.69	0.66	0.62	0.84	0.71	0.70	0.55	0.51	0.57	0.54	0.58	0.57	0.59	0.61	0.71	0.66	0.60	0.62	0
28	M	16.0	0.52	0.53	0.64	0.64	0.70	0.69	0.62	**0.58**	0.65	0.73	0.73	0.64	0.63	0.55	0.54	0.68	0.64	0.63	0.73	0.62	0.64	0.63	0.64	0.64	0.53	0.51	0.56	0.70	0.66	0.67	0.71	0.65	0.61	0.83	0.70	0.70	0.51	0.48	0.59	0.60	0.54	0.55	0.62	0.62	0.59	0.64	0.63	0.70	1
37	M	15.0	0.54	0.52	0.68	0.65	0.73	0.67	0.64	0.73	0.69	0.76	0.77	0.66	0.67	0.51	0.51	0.60	0.67	0.61	0.74	0.64	0.63	0.63	0.72	0.70	0.54	0.53	0.59	0.74	0.72	0.70	0.68	0.70	0.67	0.88	0.73	0.68	0.53	0.52	0.60	0.59	0.55	0.53	0.71	0.69	0.66	0.68	0.55	0.49	0
47	M	11.6	0.53	0.52	0.66	0.67	0.74	0.66	0.63	0.65	0.63	0.75	0.77	0.63	0.64	0.51	0.50	0.65	0.62	0.64	0.77	0.62	0.60	0.62	**0.60**	0.60	0.54	0.51	0.61	0.73	0.72	0.67	0.66	0.62	**0.59**	0.85	0.71	0.72	0.54	0.50	0.58	0.57	0.56	**0.51**	**0.57**	0.58	0.71	0.67	0.51	0.51	4
51	M	4.6	0.55	0.55	0.64	0.66	0.70	0.67	0.64	0.71	0.66	0.79	0.75	0.65	0.64	0.53	0.50	**0.39**	0.71	0.62	0.74	0.68	0.63	0.62	0.63	0.65	0.54	0.52	0.56	0.76	0.73	0.72	0.70	0.77	0.66	0.86	0.72	0.73	0.53	0.50	0.68	0.59	0.56	0.54	0.64	0.61	**0.47**	0.66	0.53	0.54	2
52	M	26.9	0.61	0.60	0.63	0.64	0.78	0.76	0.71	0.71	0.66	0.79	0.78	0.66	0.62	0.57	0.55	0.74	0.62	0.61	0.79	0.65	0.63	0.65	0.65	0.63	0.63	0.60	0.61	0.75	0.72	0.72	0.73	0.70	0.69	0.87	0.69	0.72	0.56	0.53	0.58	0.55	0.61	0.62	0.66	0.64	0.58	0.80	0.59	0.61	0
54	M	33.3	0.55	0.55	0.65	0.65	0.75	0.68	0.63	0.70	0.70	0.77	0.78	0.68	0.64	0.54	0.52	0.70	0.73	0.71	0.75	0.67	0.67	0.65	0.69	0.69	0.56	0.55	0.59	0.73	0.71	0.72	0.71	0.67	0.66	0.87	0.76	0.77	0.55	0.52	0.57	0.55	0.58	0.59	0.65	0.67	0.68	0.70	0.55	0.63	0
Mean FA (controls)			0.56	0.55	0.66	0.65	0.74	0.69	0.65	0.68	0.68	0.77	0.77	0.66	0.65	0.54	0.53	0.64	0.66	0.64	0.76	0.64	0.64	0.63	0.67	0.66	0.55	0.54	0.57	0.74	0.71	0.70	0.69	0.68	0.65	0.86	0.72	0.71	0.55	0.52	0.59	0.57	0.58	0.56	0.63	0.62	0.66	0.68	0.56	0.59	
2.5 SD threshold			0.50	0.49	0.61	0.60	0.68	0.63	0.58	0.59	0.60	0.72	0.72	0.59	0.58	0.49	0.48	0.44	0.58	0.57	0.71	0.58	0.59	0.59	0.61	0.60	0.49	0.49	0.50	0.68	0.66	0.64	0.63	0.62	0.59	0.82	0.65	0.65	0.49	0.47	0.50	0.49	0.52	0.51	0.57	0.56	0.51	0.57	0.45	0.48	
Abnormally low FA count			0	0	0	0	0	0	0	1	1	0	0	0	0	1	0	1	0	0	0	0	0	0	1	0	0	0	0	0	0	0	0	0	1	0	0	0	0	0	0	0	0	1	1	0	1	0	1	1	

Abnormally low FA values (bold typeface, yellow highlight) were defined as more than 2.5 SD below the mean of normal controls. -L: left; -R: right; ACR: anterior corona radiata; ALIC: anterior limb of internal capsule; BCC: body of corpus callosum; CGC: cingulum (cingulate gyrus); CGH: cingulum (hippocampus); CP: cerebral peduncle; CST: corticospinal tract; EC-L: external capsule; FX: fornix (column and body of fornix); FXST: fornix (cres)/stria terminalis; GCC: genu of corpus callosum; ICP: inferior cerebellar peduncle; MCP: middle cerebellar peduncle; ML: medial lemniscus; PCR: posterior corona radiata; PCT: pontine crossing tract; PLIC-L: posterior limb of internal capsule; PTR: posterior thalamic radiation (include optic radiation); RLIC: retrolenticular part of internal capsule; SCC: splenium of corpus callosum; SCP: superior cerebellar peduncle; SCR: superior corona radiata; SFO: superior fronto-occipital fasciculus; SLF: superior longitudinal fasciculus; SS: sagittal stratum (include inferior longitidinal fasciculus and inferior fronto-occipital fasciculus); TAP: tapetum; UNC: uncinate fasciculus.
